# Tilapia skin peptides, a by-product of fish processing, ameliorate DSS-induced colitis by regulating inflammation and inhibiting apoptosis

**DOI:** 10.3389/fnut.2022.988758

**Published:** 2022-09-08

**Authors:** Jiahui Gao, Lixiang Li, Dong Zhao, Xia Wang, Yanan Xia, Bing Li, Chang Liu, Xiuli Zuo

**Affiliations:** ^1^Department of Gastroenterology, Qilu Hospital, Shandong University, Jinan, China; ^2^Laboratory of Translational Gastroenterology, Qilu Hospital, Shandong University, Jinan, China; ^3^Medical Integration and Practice Center, Shandong University, Jinan, China; ^4^Robot Engineering Laboratory for Precise Diagnosis and Therapy of GI Tumor, Qilu Hospital, Shandong University, Jinan, China

**Keywords:** inflammatory bowel disease, tilapia skin peptides, apoptosis, intestinal epithelial homeostasis, LC-MS/MS

## Abstract

Intestinal bowel disease (IBD) has always been tough to treat, therefore researchers are struggle to look for treatments that are safe, low cost, and effective. Food-derived peptides are thought to have anti-inflammatory and antioxidant properties, but they have not been studied in depth in the treatment of IBD. Based on this, we explored the effect of tilapia skin peptides (TSPs) on the remission of colitis in the present study. Colonic epithelial cell lines CT-26 and HT-29 were co-treated with lipopolysaccharide (LPS) and TSPs for 12 h. Cell viability was assessed by CCK8 assay. Dextran sulfate sodium (DSS)-induced colitis model was established and 100 mg/kg TSPs were oral administered at the same time as DSS intervention. Colonic mucosal barrier function was assessed by western blotting. The inflammatory responses were evaluated by quantitative real-time PCR along with ELISA, respectively. Apoptosis was investigated by TUNEL and flow cytometry. Liquid chromatography-tandem mass spectrometry (LC-MS/MS) was used to characterize peptides composition of TSPs. It was found that TSPs significantly inhibit LPS-induced inflammation and apoptosis *in vitro* without affecting cell viability. Moreover, the upregulation and activation of Caspase-3 and Caspase-8 were also reversed by TSPs. Subsequently, *in vivo* experiments demonstrated that TSPs can attenuate DSS induced colitis, manifested by a decrease in weight loss and colon shortening. The expression of ZO-1 and occluding were significantly increased, and the pro-inflammatory cytokines were down-regulated. Meanwhile, TSPs alleviated DSS-induced apoptosis and reduced the expressions of Caspase-3 and Caspase-8. Finally, we found that TSPs were composed of 51 short peptides, and 12 of them were predicted to have significant biological activity. Collectively, this study suggested that TSPs can alleviate colon damage caused by foreign stimuli *via* inhibiting inflammation and apoptosis which indicated that it has great potential value for the treatment of IBD.

## Introduction

Inflammatory bowel disease (IBD) is a gastrointestinal inflammatory disease that encompasses ulcerative colitis (UC) and Crohn’s disease (CD), which is chronic, non-specific, recurrent and mainly involving the gastrointestinal tract (1). They’ve gotten more widespread in recent years all around the planet (2). Although the pathogenesis and mechanism of IBD remain unclear, endogenous and exogenous variables, such as immunological responses, genetic triggers, gut microbiota, and environmental effects, all work together to activate it. Epithelial erosion is a hallmark of a variety of gastrointestinal disorders, especially UC, which triggered the invasion of components of the commensal microflora and development of intestinal inflammation. Moreover, the epithelial disruption is believed to be caused by epithelial barrier dysfunction and an aberrant rise in the rate of IEC death (3, 4). Disruption of the intestinal barrier, dysregulation of cell death, and the ensuing vicious cycle of inflammation are at the heart of the chronic inflammatory progression of UC.

There are several treatment strategies for IBD. While existing treatments for UC, such as 5-aminosalicylic acid, glucocorticoids, immunosuppression therapy, and biotherapy may cause serious side effects including nephrotoxicity, acne, edema, mental instability, glucose intolerance, and dyspepsia (5). Meanwhile, current drug treatments are basically aimed at inducing remission, and there is no cure for UC presently. Accordingly, UC treatment has become a global challenge. Therefore, a safe and effective diet-based therapy is expected to be a promising option for UC treatment due to the limitations of the available therapies.

Recently, a number of investigators found that marine peptides present a broad range of biological activities including anti-inflammatory, antioxidant, antihypertensive, immunomodulatory and more (6–8). Furthermore, their low molecular weight allows them to penetrate membranes and thus function more efficiently (9). Previous researches have demonstrated that active peptides derived from dietary proteins can be delivered to target organs such as the colon to perform their functions (10). It was reported that shrimp peptide can inhibit colitis effectively in mice (11). In addition, researchers have revealed that enzymatically hydrolyzed peptides from skipjack or sturgeon all possess anti-inflammatory therapeutic benefits in murine model of colitis (12, 13). However, the currently available bioactive peptides are limited and lack relevant mechanism studies. Therefore, it is crucial to explore more safe and efficient bioactive peptides with clinical therapeutic potential.

Tilapia is one of the main freshwater fish, which contains much nutritive value to human body and is a source of high quality protein (14). About 60% of waste products, such as fish skin and bones, are produced during the fish processing, resulting in a grievous waste of resources and a certain level of pressure on the economy, society, and environment (14, 15). Therefore, it is of great significance to make full use of by-products to improve the resource utilization rate of aquatic products. Various recent studies have shown that tilapia-derived active substances are a key factor in the regulation of intestinal immunity and homeostasis. One study has shown that Tilapia skin peptides (TSPs) show a reno protective role in diabetic nephropathy by improving mitochondrial dysfunction (16). Tilapia head glycolipids were proved to protect mice from DSS-induced colitis by modulating the gut microbiota and ameliorating the gut barrier (17, 18). However, the protective effect of TSPs on DSS-induced UC mice remains unclear.

In this study, we aimed to investigate whether TSPs have protective effects against external stimuli through *in vitro* and *in vivo* experiments. And the underlying molecular mechanisms of TSPs in regulating inflammation and inhibiting apoptosis were also evaluated. This is the first time to evaluate the protective effect of TSPs on DSS-induced colitis, and characterize the peptides composition of TSPs by mass spectrometry.

## Materials and methods

### Preparation of tilapia skin peptides

The TSPs were purchased from Hainan Pure Peptide Technology Co., Ltd. (Haikou, China). The production process was as follows: tilapia skin was washed and then hydrolyzed at a high temperature of 100°C for 1–2 h, then it was hydrolyzed sequentially with neutral and alkaline protease at 50°C for 90 min under the conditions of pH 7.0 and pH 9.0, respectively. Next, the liquid was filtered through a double filter and a plate-and-frame filter, respectively, to remove the suspended solids and residues to obtain a clear liquid. The filtered liquid was passed through a 0.22 μm filter screen to remove viable bacteria and then vacuum concentrated to a solid content of 35–45%. Finally, the liquid was spray-dried into powder for subsequent experiments.

### Cell culture and treatment

CT-26 and HT-29 cell lines were purchased from the American Type Culture Collection (Manassas, VA, United States). CT-26 was incubated in Roswell Park Memorial Institute-1640 (RPMI1640; GIBCO, Rockville, MD, United States) and HT-29 was cultured in Dulbecco’s Modified Eagle Medium (DMEM; GIBCO, Rockville, MD, United States) medium supplemented with 10% FBS (invigentech, CA, United States), 100 U/mL penicillin and 100 μg/mL streptomycin, respectively. The two cells were incubated in a 37°C, 5% CO2 cell incubator. To detect the bioactive effect of TSPs, we seeded cells into 96-well plates for CCK8 assay, immunofluorescence; and 6-well plates for protein and RNA extraction and flow cytometry analysis. The cells were treated differently as follows: (1) Control groups: without TSPs incubated and LPS stimulated; (2) LPS groups: cells were incubated in the absence of TSPs with lipopolysaccharide (LPS, 1 μg/mL, Sigma-Aldrich) stimulation for 12 h; (3) LPS + TSPs groups: cells were treated with TSPs (50 ng/mL) and LPS (1 μg/mL) simultaneously for 12 h.

### Cell counting kit-8 assay

CT-26 and HT-29 colonic epithelial cells were seeded in 96-well and treated with or without TSPs (50 ng/ml) for 12 h. Using the Cell Counting Assay-8 kit (CCK-8; Beyotime Biotechnology, Shanghai, China), the relative numbers of viable cells were determined. After the cells adhered sufficiently, 20 μl of CCK-8 was then added to each well and incubated for 1 h. Then the results were recorded in absorbance optical density at 450 nm.

### Flow cytometry

Flow cytometry was used to detect cell apoptosis using the annexin V-FITC apoptosis detection kit (BestBio, Shanghai, China), as directed by the manufacturer’s instructions. Adherent cells were digested by trypsin without EDTA. Discard the medium after centrifugation at 300 × g for 5 min at 4°C. Cells were washed twice with cold PBS. Resuspend cells with 400 μl of 1X Annexin V Binding Solution at a concentration of approximately 1 × 106 cells/ml. Add 5 μl Annexin V-FITC staining solution to the cell suspension, mix gently and incubate at 4°C for 15 min in the dark. 5 μl PI staining solution was added and incubated for 5 min. Immediately detected and analyzed early and late apoptosis of cells by Beckman Coulter Gallios flow cytometer.

### Animals and induction of colitis

Male C57BL/6J mice, 7–8 weeks old, were purchased from (Beijing SPF Biotechnology Co., Ltd., China), 4 mice per cage were housed in a standard SPF facility of Shandong University at 22°C, 12-h day and night cycle. 3% DSS (MP Biologicals, Solon, OH, United States) was administered through drinking water to induce colitis.

### Treatments and sample collection

The C57BL/6J mice were randomly assigned into three groups (8 mice per group) after 1 week of acclimation. All groups were free to drink water. Figure 5A depicts the experimental design.

The experimental design is as follows: (1) Control group: 7 days of tap water and daily oral gavage of PBS; (2) DSS group: 3% DSS and daily oral gavage of PBS for 7 days; (3) DSS + TSPs group: 3% DSS and daily oral gavage of 100 mg/kg TSPs for 7 days. For the entire duration of the trial, body weight was measured daily. On day 7, the mice were sacrificed after anesthesia followed by measuring the length of the colon. After rinsing the tissues with cold PBS, they were snap frozen in liquid nitrogen for subsequent experiments. For histological investigation, a 5 mm segment of the colon was washed with PBS and then fixed in 4% paraformaldehyde.

### Histological analysis of the colon

The collected colon tissues were fixed in 4% paraformaldehyde for 24 h and then dehydrated. Next, they were paraffin-embedded and made into 4 μm tissue slices, stained with hematoxylin and eosin. Referring to previous studies, slices were assessed blindly, and the scores were evaluated according to the following parameters: inflammatory infiltration, crypt structure, ulceration and with or without edema (19).

### Ribonucleic acid extraction and quantitative real-time polymerase chain reaction (qRT-PCR)

After extraction of total RNA from mouse colon tissues and cultured cells with the TRIzol reagent (Invitrogen, CA, United States), the ReverTra Ace qPCR RT kit (Toyobo, Osaka, Japan) was used for the reverse transcription single-stranded cDNA. For quantitative PCR amplification, M5 Super qPCR RT kit with gDNA remover (SYBR Green) (Mei5 biotechnology, Beijing, China) was utilized. Table 1 list the gene-specific primers used in PCR, which were performed in a 20-μL system. The data was obtained and the expression level of RNA was calculated in the 2-ΔΔCt method.

**TABLE 1:** Primer sequence for qPCR.

	Forward	Reverse
miceIL-1β	GAAATGCCACCT TTTGACAGTG	TGGATGCTCT CATCAGGACAG
miceIL-6	CTGCAAGAGA CTTCCATCCAG	AGTGGTATAGA CAGGTCTGTTGG
miceCASP3	GGAGTCTGACTG GAAAGCCGAA	CTTCTGGCAAG CCATCTCCTCA
miceCASP8	ATGGCTACGGT GAAGAACTGCG	TAGTTCACGCC AGTCAGGATGC
miceIL-17A	CAGACTACCTCA ACCGTTCCAC	TCCAGCTTTCC CTCCGCATTGA
miceTNF-α	CAGGCGGTGC CTATGTCTC	CGATCACCCCGA AGTTCAGTAG
miceGAPDH	CATCACTGCCAC CCAGAAGACTG	ATGCCAGTGAGC TTCCCGTTCAG
humanIL-1β	AGCTACGAATC TCCGACCAC	CGTTATCCCATG TGTCGAAGAA
humanIL-6	ACTCACCTCTTCA GAACGAATTG	CCATCTTTGGAAGG TTCAGGTTG
humanCASP3	GGAAGCGAATCA ATGGACTCTGG	GCATCGACATCTG TACCAGACC
humanCASP8	AGAAGAGGGTCA TCCTGGGAGA	TCAGGACTTCCT TCAAGGCTGC
humanGAPDH	GTCTCCTCTGACTTC AACAGCG	ACCACCCTGTTGCTG TAGCCAA

### Enzyme-linked immunosorbent assay

The total protein was extracted by RIPA lysis buffer (Solarbio, Beijing, China) from colon tissue. A bicinchoninic acid (BCA) protein assay kit (ABP Biosciences, Rockville, United States) was used to evaluate the protein content in accordance with the manufacturer’s instructions. Pro-inflammatory cytokines in colon tissues were quantified using an ELISA kit (Lianke Biotechnology, Shanghai, China) according to the manufacturer’s instructions. The cell culture concentrations of pro-inflammatory cytokines were also measured similarly.

### Western blotting analysis

Protein extraction and concentration determination were performed as above. SDS-PAGE was used to separate the proteins in the sample by molecular weight in the gel, and then the proteins were transferred to PVDF membranes, which were blocked with 2.5% skim milk for 1 h at room temperature, and then the PVDF membranes were incubated with primary antibodies overnight. The primary antibodies used in this experiment: ZO-1 Polyclonal Antibody (1:1,000, “abs131,224”; Absin, Shanghai, China), Occludin Polyclonal Antibody (1:1,000, “abs136,990”; Absin, Shanghai, China), Caspase-3 antibody (1:1,000, “9,662”; Cell Signaling Technology, Danvers, United States), Caspase-8 Polyclonal antibody (1:500, 13423-1-AP; Proteintech, Wuhan, China). After the primary antibody incubation, membranes were washed three times with TBST for 10 min each. Then they were incubated with HRP-conjugated secondary antibody diluted in 2.5% skim milk for 1 h at room temperature. Signals were visualized through the electrochemiluminescence (ECL) detection system. Protein fluorescence density and relative expression were evaluated by Image J software (NIH, Bethesda, United States).

### TUNEL assay

According to the notch end labeling approach, the formalin-fixed colonic sections were fluorescently stained as directed by the TUNEL Apoptosis Detection Kit (Green Fluorescence, Abbkine, Wuhan, China). After deparaffinized and rehydrated, the tissue sections were incubated in 20 μg/mL proteinase k for 15 min before being stained with TUNEL staining reagent in the dark for 2 h, and images were taken with a fluorescence microscope.

TUNEL labeling was used *in vitro* to detect the apoptosis index as well. Briefly, cells were fixed with 4% paraformaldehyde for 30 min and washed three times with PBS for 5 min each. Added 50 μL of 0.3% Triton X-100 to each well and incubated for 30 min to permeabilized cells. Then, the cells were incubated with 50 μL of TUNEL reaction solution at 37°C for 2 h. Finally, the images were observed with a fluorescence microscope and Image J software (NIH, Bethesda, United States) was used to calculate the proportion of positively stained total cells.

### Immunohistochemistry staining

Colon tissues were fixed and embedded with formalin, and 4 μm thick sections were cut. After deparaffinizing, rehydrating, 0.1 M citrate buffer was used for antigen recovery. These slices were then blocked the non-specific binding following soaking in goat serum for 30 min. They were then incubated with primary Caspase-3 antibody (1:1,000, “9,662”; Cell Signaling Technology, Danvers, United States), Caspase-8 Polyclonal antibody (1:100, 13423-1-AP; Proteintech, Wuhan, China). After three 5-min washes with PBS, the second antibody was put on slides and incubated for 30 min. Then DAB and hematoxylin were used to stain, respectively. Mean of IOD was evaluated using the Image-Pro (^®^) Plus software.

### LC-MS/MS analysis and peptides identification

The proteins of the samples were separated by gel electrophoresis, the samples were cut into 1 mm particles, and the peptides were obtained by enzymatic hydrolysis with 0.01μg/μL trypsin, and then frozen and drained. After being redissolved in mobile phase A (2% ACN, 0.1% FA), the peptide samples were centrifuged for 10 min at 20,000 g, and then were injected. Liquid phase separation was performed by a nano liter liquid chromatograph (Eksigent Ultra 2D, SCIEX, Framingham, MA, United States). After separation, the peptides were transferred to ESI tandem mass spectrometer (Triple TOF 5600, SCIEX, Framingham, MA, United States). Scanning was set as high sensitivity mode. Fragmentation Energy selected “RollingCollision Energy”. The analyzed data were searched against the protein database of Tilapia for peptide matching.

### *In silico* prediction of the identified peptides

Sequences of the peptides were inputted into the PeptideRanker web server to assess their potential biological activity, which using the N-to-1 neural network probability^1^ (Mooney, Haslam, Pollastri, and Shields, 2012). The assigned scores ranged from 0 to 1.0 and any peptide predicted to have a threshold greater than 0.5 was classified as bioactive according to the database.

### Statistical analysis

All data was expressed as means ± SD and analyzed with the GraphPad Prism 8.0 (GraphPad Software, San Diego, Canada). The differences between two groups were analyzed using the Student’s *t*-test. To compare data from more than two groups, one-way ANOVA was utilized. If the adjusted *p*-value was less than 0.05, it was considered statistically significant.

## Results

### TSPs prevented lipopolysaccharide-induced inflammation and apoptosis in mice colonic epithelial cell line CT-26

**FIGURE 1:**
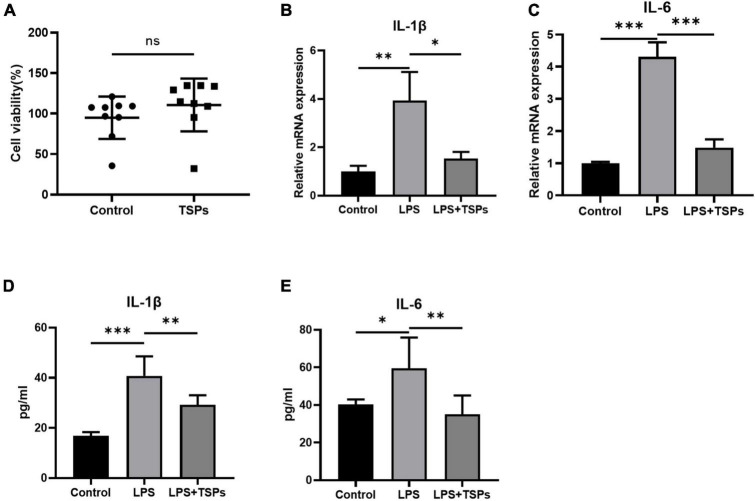
The cell viability and expression of pro-inflammatory cytokines of mice colonic epithelial cell line CT-26. **(A)** Cell viability was determined by CCK-8 assay after TSPs treatment (*n* = 9). **(B,C)** Relative mRNA expression of two pro-inflammatory cytokines, IL-1β and IL-6 (*n* = 3). The concentrations of IL-1β **(D)**, IL-6 **(E)** measured by ELISA (*n* ≥ 5). Data were presented as means ± SD. Statistical significance was determined using Student’s *t*-test or one-way ANOVA. *P < 0.05, **P < 0.01, ***P < 0.001; ns, no significance.

To explore the potential bioactivity of TSPs, the effects of TSPs on LPS-induced inflammation and apoptosis were detected in colonic epithelial cell line CT-26 which derived from mice. First, the TSPs did not affect cell viability based on CCK8 assay (Figure 1A). Then, the CT-26 cells were incubated with 50 ng/ml TSPs concurrently stimulated with 1 μg/ml LPS. We found that treatment of CT-26 cells with TSPs could obviously abolish LPS-induced upregulation of IL-1β and IL-6 in transcript levels (Figures 1B,C). At the protein levels, TSPs also had the ability to down-regulate the pro-inflammatory cytokines in agreement with the transcription levels, indicating that TSPs exert an anti-inflammatory property in the vitro (Figures 1D,E).

**FIGURE 2:**
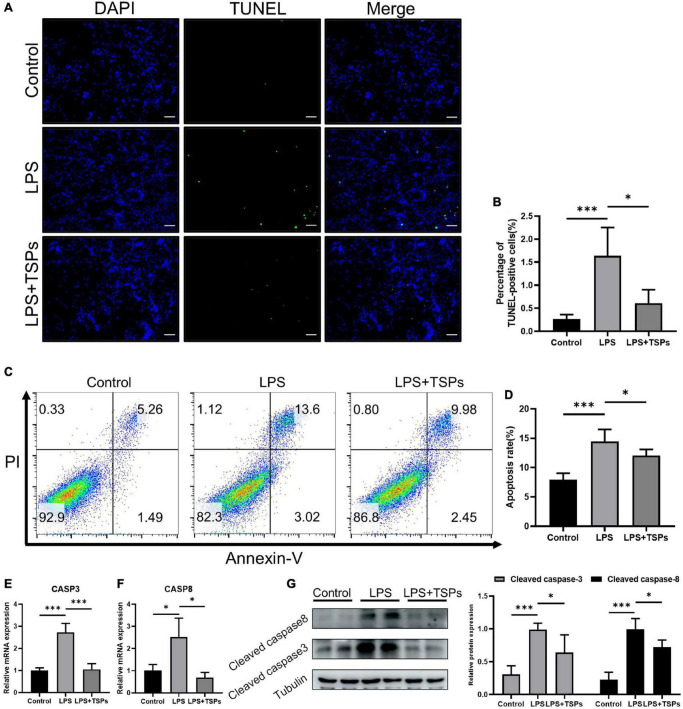
TSPs prevented LPS-Induced Apoptosis in CT-26. **(A)** Representative images of TUNEL staining in CT-26 cells. Scale bar = 100μm. **(B)** Quantification of the percentage of TUNEL positive cells (*n* = 6). **(C)** Apoptosis analysis of CT-26 cells by flow cytometry. **(D)** The results of quantitative analyses of apoptosis rate (*n* = 5). **(E,F)** Caspase-3 and caspase-8 levels were measured by qRT-PCR (*n* = 3). **(G)** Western blot analysis of the cleaved caspase-3 and cleaved caspase-8 proteins in CT-26 cells and semi-quantitative analysis of relative indicated proteins expression (*n* = 4). Data were presented as means ± SD. Statistical significance was determined using one-way ANOVA. *P < 0.05, ***P < 0.001.

The intestinal epithelial cell apoptosis was detected by two methods. The 3′-OH terminus of fragmented DNA in apoptotic cells can be measured by TUNEL staining under the action of deoxyribonucleotide terminal transferase. In addition, annexin-V detects the phosphatidylserine during apoptosis and PI makes the nucleus red-stained through the cell membrane of the middle and late stages of apoptosis and dead cells (20, 21). As shown in Figures 2A,B, the number of apoptotic cells increased notably following LPS treatment and was down-regulated by TSPs. And these results were confirmed by flow cytometry analysis which showed that the increased rate of apoptotic cells after LPS administration declined sharply after TSPs treatment (Figures 2C,D). Furthermore, caspase-3 and caspase-8 were central molecules of apoptosis pathway and played an important role in it (22). We observed that the mRNA of caspase-3 and caspase-8 stimulated by LPS significantly decreased after TSPs treatment (Figures 2E,F). Similarly, western blot analysis presented that TSPs marked reduced cleaved caspase-3 and cleaved caspase-8 expression (Figure 2G).

Together, these results proved that TSPs exert significant therapeutic effects against LPS-induced inflammation and apoptosis in mouse colonic epithelial cell lines. These mechanisms could be related to the protection of intestinal epithelial lesions caused by LPS.

### Tilapia skin peptides relieved lipopolysaccharide-induced inflammation and apoptosis in human colonic epithelial cell line HT-29

**FIGURE 3:**
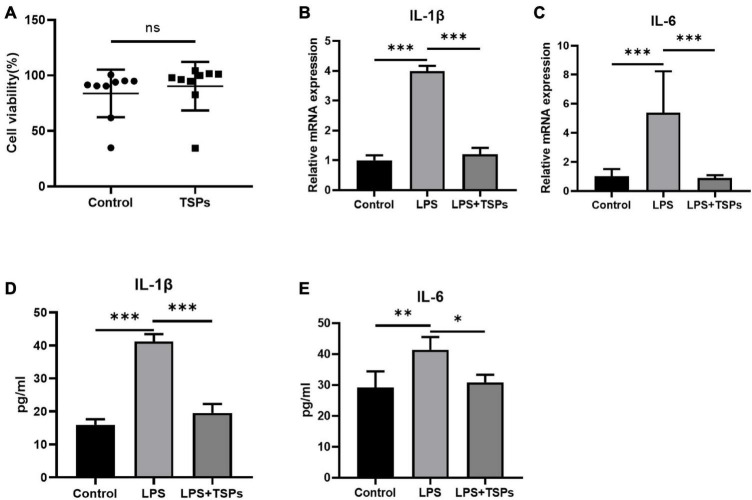
The cell viability and expression of pro-inflammatory cytokines of human colonic epithelial cell line HT-29. **(A)** Cell viability was determined by CCK-8 assay after TSPs treatment (*n* = 9). **(B,C)** Relative mRNA expression of two pro-inflammatory cytokines, IL-1β and IL-6 (*n* ≥ 3). The concentrations of IL-1β **(D)**, IL-6 **(E)** measured by ELISA (*n* = 6). Data were presented as means ± SD. Statistical significance was determined using Student’s *t*-test or one-way ANOVA. *P < 0.05, **P < 0.01, ***P < 0.001; ns, no significance.

Given that researches on TSPs in human cells or tissues are more conducive to future implications on human diseases therapy, we further explored the impact of TSPs on the inflammation and apoptosis of LPS-treated HT-29 cell line. The effect of TSPs on cell proliferation was first investigated. After co-culturing with TSPs for 12 h, HT-29 cells viability unchanged compared with control groups (Figure 3A). To assess the inflammatory response, we detected the mRNA levels of pro-inflammatory cytokines such as IL-1β and IL-6 in response to LPS stimulation and found that they are markedly decreased after TSPs treated (Figures 3B,C). In line with the mRNA levels, the protein levels of IL-1β and IL-6 significantly decreased in the TSPs-treated group compared with the LPS group detected by ELISA assay (Figures 3D,E), confirming that TSPs still exhibit significant biological activity in the human-derived cell model.

**FIGURE 4:**
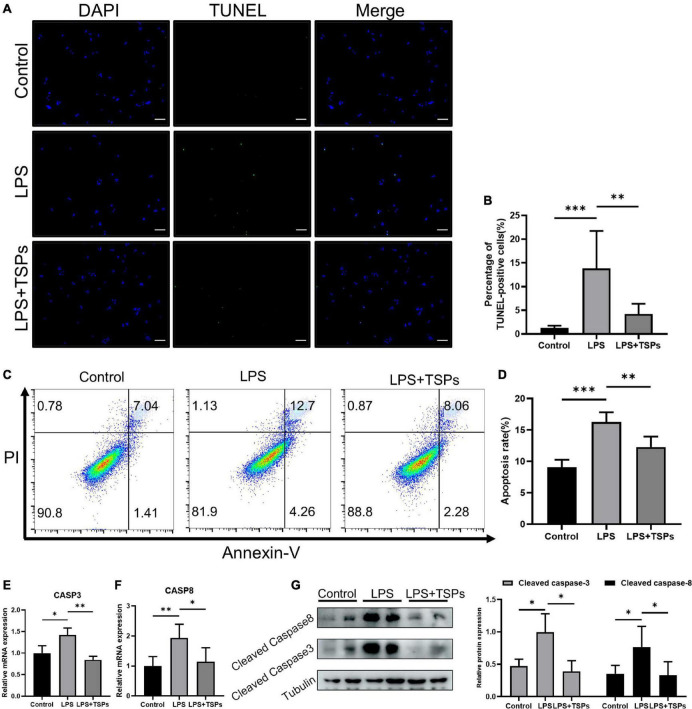
TSPs prevented LPS-Induced Apoptosis in HT-29. **(A)** Representative images of TUNEL staining in HT-29 cells. Scale bar = 100μm. **(B)** Quantification of the percentage of TUNEL positive cells (*n* = 6). **(C)** Apoptosis analysis of HT-29 cells by flow cytometry. **(D)** The results of quantitative analyses of apoptosis rate (*n* = 5). **(E,F)** Caspase-3 and caspase-8 levels were measured by qRT-PCR (*n* ≥ 3). **(G)** Western blot analysis of the cleaved caspase-3 and cleaved caspase-8 proteins in HT-29 cells and semi-quantitative analysis of relative indicated proteins expression (*n* = 5). Data were presented as means ± SD. Statistical significance was determined using one-way ANOVA. *P < 0.05, **P < 0.01, ***P < 0.001.

Then, we used TUNEL and Annexin V-FITC/PI staining assays to assess the LPS-induced apoptosis of HT-29 cells when treated with TSPs or not. As shown in Figures 4A,B, fluorescence intensity in TUNEL assays was stronger in LPS group, indicating a higher rate of apoptosis. Whereas administration of TSPs reduced the immunofluorescence staining and apoptosis rate of HT-29 cells to some extent. According to flow cytometry studies, the inhibitory effect of TSPs on apoptosis was confirmed again (Figures 4C,D). Consistently, qPCR and western blot analysis also revealed that the expression and activation of caspase-3 and caspase-8 were also suppressed after TSPs treatment (Figures 4E–G). Altogether, these data suggested that in human-derived cell lines, TSPs are also useful in regulating LPS-induced inflammation and apoptosis.

**FIGURE 5:**
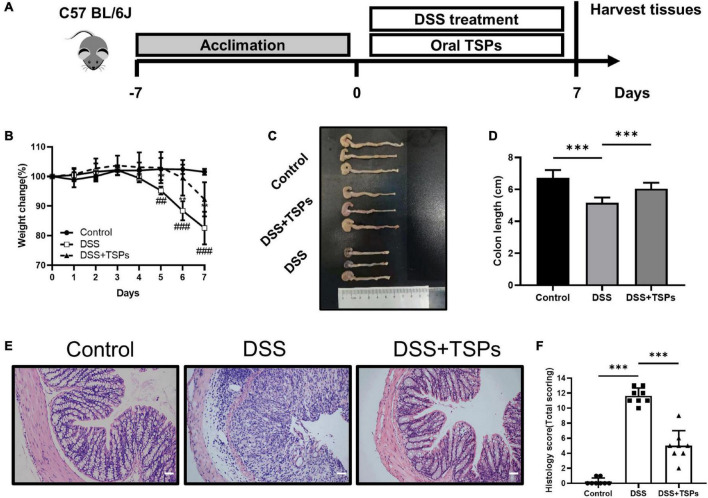
Oral TSPs administration alleviated DSS-induced colitis. **(A)** Graphical description of experimental design of this study. **(B)** Daily body weight changes following DSS treatment (*n* = 8). Statistical significance was determined using two-way ANOVA. **P < 0.01 relative to DSS group; ^##^*P* < 0.01, ^###^*P* < 0.001 relative to Control group. **(C)** Macroscopic pictures of colons and **(D)** the statistical colon length from different groups (*n* = 8). **(E)** H& E stained colon sections and **(F)** histological scores of colons (*n* = 8). Data were presented as means ± SD. Statistical significance was determined using one-way ANOVA. *P < 0.05, **P < 0.01, ***P < 0.001.

### Oral tilapia skin peptides administration alleviated DSS-induced colitis

Given that TSPs have distinct inhibitory effects on inflammation and apoptosis in cell lines *in vitro*, we next sought to determine whether TSPs exert beneficial effects *in vivo*. Mice were given 3% DSS in water continuously which caused them to develop experimental colitis and daily oral 100 mg/kg body weight TSPs at the same time for 7 days to evaluate the alleviating effect of TSPs on DSS-induced colitis. On day 0, the initial body weight of all mice was essentially the same. In the control group, the body weight did not change significantly. During the 7 days of DSS intervention, oral TSPs significantly decreased body weight loss compared with the DSS group (Figure 5B). Colon length and morphological changes are indicators of intestinal inflammation. DSS made the colon edema, erosion, thinner, and shorter compared with control group. However, TSPs intervention markedly relieved these symptoms (Figures 5C,D). HE staining showed normal mucosal structure in control group. In turn, histological analysis of DSS group displayed more severe inflammatory cell infiltration, mucosal damage, goblet cell deficiency and a higher overall histology score (Figures 5E,F). After TSPs intervention, mucosal inflammation symptoms improved significantly such as glands arranged neatly and there was less neutrophil and lymphocyte infiltration (Figure 5E). In addition, after treatment with TSPs, the histological score decreased significantly (Figure 5F).

Altogether, these data indicated that oral TSPs can alleviate clinical colitis symptoms and colonic damage triggered by DSS.

### Oral tilapia skin peptides improved the mucosal barrier function and inhibited the expression of inflammatory cytokines

**FIGURE 6:**
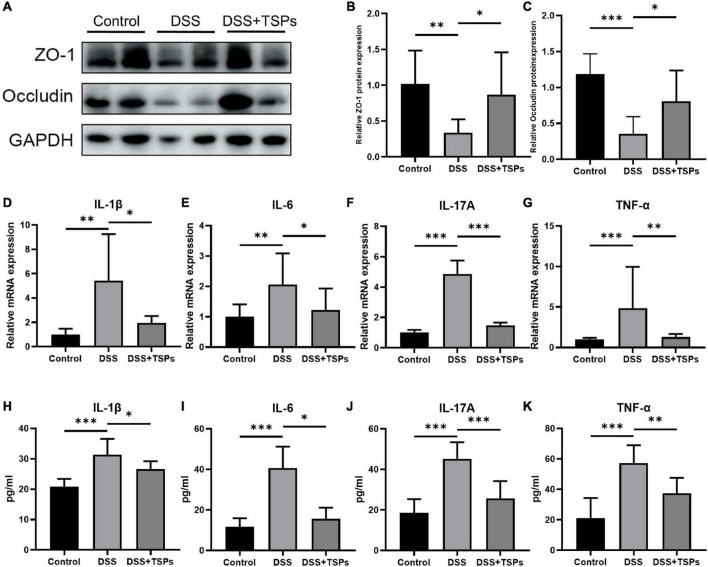
Oral TSPs improved the mucosal barrier function and inhibited the expression of inflammatory cytokines. **(A)** Western blot analysis of the tight junction proteins ZO-1, occludin in the colon. Grayscale statistics of ZO-1 **(B)** and occluding **(C)** relative to internal control (*n* = 8). **(D–G)** Relative mRNA expression of pro-inflammatory cytokines, IL-1β, IL-6, IL-17A and TNF-α (*n* = 8). The concentrations of IL-1β **(H)**, IL-6 **(I)**, IL-17A **(J)** and TNF-α **(K)** measured by ELISA in the colon (*n* = 8). Data were presented as means ± SD. Statistical significance was determined using one-way ANOVA. *P < 0.05, **P < 0.01, ***P < 0.001.

The increase in intestinal permeability caused by impaired intestinal barrier function is one of the pathogenesis of intestinal inflammation (23, 24). Therefore, we further detected the intestinal barrier function in order to explore the specific protective mechanism of TSPs against DSS colitis. Western blot density analysis presented the expression of ZO-1 and occludin was substantially down-regulated after DSS intervention. Of note, oral TSPs significantly increased these tight junction proteins expression in colon tissue compared with DSS group (Figures 6A–C).

To more precisely determine how TSPs affect colonic inflammatory responses, the expression of pro-inflammatory cytokines in colonic tissues were measured. Colon IL-1β, IL-6, IL-17A and TNF-α mRNA levels were considerably increased in DSS treated groups but decreased significantly in response to oral TSPs (Figures 6D–G). Moreover, TSPs considerably decreased IL-1β, IL-6, IL-17A and TNF-α concentrations in protein level by ELISA compared with DSS group (Figures 6H–K). Overall, these evidences suggested that TSPs can attenuate intestinal damage by enhancing intestinal barrier function and inhibiting the expression of pro-inflammatory cytokines.

### Oral tilapia skin peptides reduced DSS-induced apoptosis

**FIGURE 7:**
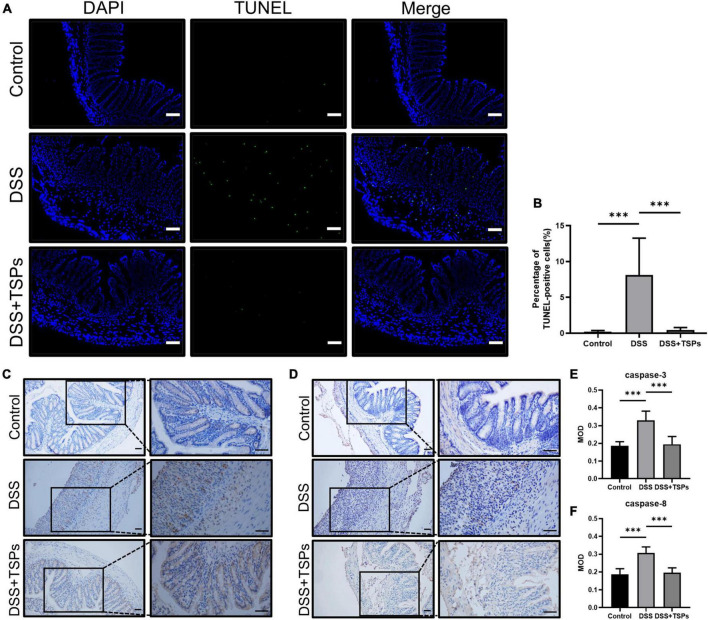
Oral TSPs reduced DSS-induced apoptosis. **(A)** Immunofluorescence TUNEL staining of colonic sections. Scale bars = 50 μm. **(B)** Apoptosis rate in colonic sections (*n* = 8). **(C)** Representative images of immunohistochemistry staining for caspase-3 protein. Scale bar = 50 μm. **(D)** Representative images of immunohistochemistry staining for caspase-8 protein. Scale bar = 50 μm. **(E,F)** Immunohistochemical image gray-scale analysis of the caspase-3 and caspase-8 of the mean of density (MOD) (*n* = 8). Data were presented as means ± SD. Statistical significance was determined using one-way ANOVA. ***P < 0.001.

It is well known that there is a dramatic accumulation of apoptotic cells in the colonic mucosa with the aggravation of colonic mucosa inflammation. And dysregulated or excessive apoptosis might result in serious intestinal diseases. TUNEL analysis showed that there was more positive staining in the colon section of DSS group, but TSPs significantly reduced the amount of green fluorescence in the colonic mucosa compared to DSS-treated mice (Figure 7A). Moreover, the scientific count on the proportion of apoptotic cells was consistent with what we observed (Figure 7B). Next, we further measured the caspase-3 and caspase-8 expressions in the colon of DSS-induced mice with or without TSPs treatment by immunohistochemical staining, which showed clear increased staining in the colonic mucosa of DSS-induced colitis mice compared with controls, while TSPs intervention largely reversed this trend (Figures 7C–F). In conclusion, TSPs were shown to significantly modulate the apoptosis signaling pathway and reduce the expression of certain caspases in DSS mice, which may be one of the mechanisms by which they alleviate intestinal injury.

### Characterization of tilapia skin peptides

Previous studies have proved that foodborne bioactive peptides have a variety of biological effects, although the potential mechanisms are not clear, the special amino acid sequence and composition of peptides is considered to be one of the unique mechanisms (25). As a result, to identify distinct peptides, TSPs were submitted to LC-MS/MS analysis. Consequently, we discovered 51 peptide sequences in TSPs which had 6–26 amino acids in length. The sequences, modification and protein origin of the identified peptides were listed in Table 2. There were 12 peptides derived from the C1q domain-containing protein, which has been discovered in the C-terminus of vertebrate secreted or membrane-bound proteins, predominantly short-chain collagens and collagen-like structures, and consists of roughly 136 amino acids. It was suggested that most of the peptides produced by enzymatic hydrolysis were derived from tilapia collagen protein. Besides, other peptides were originated from proteins like SERPIN domain-containing protein, Fibrillar collagen NC1 domain-containing protein or IF rod domain-containing protein and so on.

**TABLE 2:** The identified peptides of TSPs.

Peptide	Modification	Protein origin
GPTGPQGPAGPQGLK	Deamidated (NQ)@12Q	C1q domain-containing protein
SPMSAFTAALTTPYPPAGSPIK	Oxidation (M)@3M	C1q domain-containing protein
SPMSAFTAALTTPYPPAGSPIK	–	C1q domain-containing protein
NEEPVLFTYDEYNK	Deamidated (NQ)@1N; deamidated (NQ)@13N	C1q domain-containing protein
NEEPVLFTYDEYNK	Deamidated (NQ)@1N	C1q domain-containing protein
NEEPVLFTYDEYNK	–	C1q domain-containing protein
NEEPVLFTYDEYNK	Deamidated (NQ)@13N	C1q domain-containing protein
GPQGPAGQPGSPGMPGVGK	Oxidation (M)@14M	C1q domain-containing protein
NGQPVMFTYDEYNK	Oxidation (M)@6M	C1q domain-containing protein
NGQPVMFTYDEYNK	–	C1q domain-containing protein
NGQPVMFTYDEYNK	Deamidated (NQ)@3Q	C1q domain-containing protein
GFLDEMAGSAVLQLYTGDR	Oxidation (M)@6M	C1q domain-containing protein
GFLDEMAGSAVLQLYTGDR	–	C2q domain-containing protein
DDGNHATVLLLPYK	–	SERPIN domain-containing protein
ESGITDAFGDK	–	SERPIN domain-containing protein
LSAPNADFAVVLYK	–	SERPIN domain-containing protein
TAAGNNIFFSPLGISTALSLLSTGAR	–	SERPIN domain-containing protein
LDVGNAAAVR	–	SERPIN domain-containing protein
GETGPVGATGPSGPQGSR	–	Fibrillar collagen NC1 domain-containing protein
GEGGSFGPAGPAGPR	–	Fibrillar collagen NC1 domain-containing protein
FASFIDK	–	IF rod domain-containing protein
LALDIEIATYR	–	IF rod domain-containing protein
EAYPGDVFYLHSR	–	ATP synthase subunit alpha
VIQCSDLGLK	Carbamidomethyl (C)@4C	Decorin
QASTADISI	Deamidated (NQ)@1Q	Fibrinogen C-terminal domain-containing protein
VGINGFGR	–	Glyceraldehyde-3-phosphate dehydrogenase, EC 1.2.1.12
VIENLTVLK	Deamidated (NQ)@4N	HEPN domain-containing protein
VATLQAQLEQSRR	Deamidated (NQ)@5Q; deamidated (NQ)@7Q	Myosin motor domain-containing protein
QDWHPFLPK	–	Phospholipid scramblase
TVLTQEALISVK	Deamidated (NQ)@5Q	PRELI/MSF1 domain-containing protein
EDIGQLVLGAR	Deamidated (NQ)@5Q	Si:ch211–129c21.1
LAADDFR	–	Si:ch211–156l18.7
LEQEIATYR	–	Si:ch211–156l18.7
QKMVEEER	Gln->pyro-Glu (N-term Q)@N_term; deamidated (NQ)@1Q	Wu:fc23c09
SCTLDGQVFADR	Carbamidomethyl (C)@2C	Uncharacterized protein
GFPGLPGPSGEPGK	–	Uncharacterized protein
VRVPLLVK	–	Uncharacterized protein
GIVGLPGQR	–	Uncharacterized protein
GEAGAVGVAGPSGPR	–	Uncharacterized protein
TFGSCTLDGQLYNDK	Carbamidomethyl (C)@5C	Uncharacterized protein
TFGSCTLDGQLYNDK	Carbamidomethyl (C)@5C; deamidated (NQ)@10Q; deamidated (NQ)@13N	Uncharacterized protein
VLIELARR	–	Uncharacterized protein
MAAVPGVR	–	Uncharacterized protein
GESGPSGPAGPAGPAGVR	–	Uncharacterized protein
KVLMVGPR	–	Uncharacterized protein
SVTLSGIR	–	Uncharacterized protein
ILIPLR	–	Uncharacterized protein
LPILIDSPK	–	Uncharacterized protein
IATALDPR	–	Uncharacterized protein
GETGPAGPAGAAGPAGPR	–	Uncharacterized protein
IQQSLTSGNLFTVK	Deamidated (NQ)@3Q	Uncharacterized protein

Each peptide was scored and its biological activity was evaluated *in silico*. Table 3 present the scores (only above 0.5) and sequences for the peptides with the potential to exert biological activity, respectively. This result suggested that the following 12 peptides may play a relatively more important role in this study.

**TABLE 3:** Activity score results of TSPs.

Peptide sequence	Score
QDWHPFLPK	0.916566
GESGPSGPAGPAGPAGVR	0.855864
GPTGPQGPAGPQGLK	0.837251
GFPGLPGPSGEPGK	0.83496
GPQGPAGQPGSPGMPGVGK	0.770195
GETGPAGPAGAAGPAGPR	0.731973
GEGGSFGPAGPAGPR	0.711084
FASFIDK	0.630491
VGINGFGR	0.612399
ILIPLR	0.571373
LAADDFR	0.544243
LSAPNADFAVVLYK	0.503917

## Discussion

**FIGURE 8:**
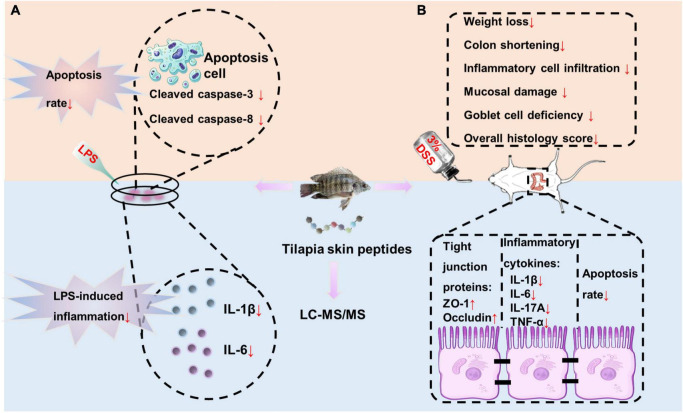
Overview of TSPs inhibiting inflammation and apoptosis caused by external stimuli. **(A)** TSPs relieved LPS-induced inflammation and apoptosis *in vitro*. **(B)** TSPs alleviated DSS-induced inflammation and apoptosis *in vivo*.

In the present study, we characterized a novel type marine peptide derived from tilapia skin by LC-MS/MS for the first time and elucidated its effect on DSS-induced colitis. Our findings indicated that TSPs protect against the damage by inhibiting inflammation and apoptosis induced by external stimuli (Figure 8). This is the first evidence had been presented for the effect of TSPs in DSS colitis and their peptides composition had been characterized. Our findings offer a new insight into the mechanism of marine peptides action, as well as a novel strategy for treatment of gut inflammation-associated diseases.

Cytokines produced by the intestinal mucosa have a significant impact on immunological outcomes. In the mucosa of UC, the production of pro-inflammatory cytokines is excessive, with more non-specific inflammatory cells flowing into the mucosa, inducing immune response and inflammation (26). Therefore, strategies aimed at inhibiting downstream inflammatory processes should have therapeutic effects for IBD. Many anti-cytokine antibodies, cytokines or cytokine modifiers have been reported for the effective therapeutic targets of UC (27). Here, we reported that treatment with TSPs at the same time as LPS intervention decreased the production of proinflammatory cytokines in both mice and human colonic epithelial cell lines and it was confirmed in DSS-induced colitis *in vivo*. This is consistent with a previous experiment proved that fish hydrolysates derived from marine byproducts reduced the expression of IL-1β and IL-6 in LPS-induced neuroinflammation mice (28). In addition to their potential direct damage to the gut, pro-inflammatory cytokines activate downstream mechanisms of action that cause important pathophysiological changes. Therefore, the subsequent in-depth mechanism of TSPs action on colonic mucosa needs to be further studied.

The gut inflammation, gut physical barrier and gut cell death have a complex interaction with each other in order to maintain the intestinal homeostasis. If one of them is disrupted or altered, the others may change as well (4). Since we observed that TSPs significantly regulate inflammation in the previous study, it is speculated that TSPs have a corresponding effect on cell death patterns. The most common and well-studied type of inflammation-induced cell death is apoptosis (29). Although programmed cell death may play a beneficial part in inflammation, excessive cell death leads to the release of a great deal of intracellular pro-inflammatory factors, amplifying the cascade of inflammation and injury that causes inflammation (30, 31). Intestinal epithelial cell apoptosis is considered to be an early event in the development of UC and plays a vital role in it (32). It has been reported that epithelial cell loss in active UC is primarily caused by apoptosis in the crypts of both involved and uninvolved areas (33). Therefore, effective inhibition of epithelial cell apoptosis is of great significance for the clinical treatment of UC, and provides a possible direction for the research and development of therapy agents. This study demonstrated that the increase of apoptosis rate in LPS treated cell lines and DSS-mice colon were reversed after TSPs treatment either by annexin V-FITC detection or TUNEL assay, indicating that TSPs possess a protective effect on apoptosis-induced damage and thereby reduce intracellular harmful factors release and alleviate inflammation. This was consistent with previous studies reporting that marine-derived bioactive peptides have anti-apoptotic effects (34, 35). Inhibition of apoptosis clearly indicated a reduction in the degree of colonic injury and may be one of the mechanisms by which TSPs exert their functions.

Next, we further explored the mechanism of TSPs inhibiting apoptosis. Caspases, which as cleaving cellular proteins required for the breakdown of dying cells, are responsible for apoptotic cell death (36). According to previous studies, regulating the expression of caspase as well as inhibiting its activation may be a strategy for alleviating colitis. And it has been previously reported that betulin attenuated acetic acid-induced UC by inhibiting the expression of caspase-3 and caspase-8 in the colon (37). In consequence we then explored the difference of expression and activation of caspase-3 and caspase-8 between several groups. As expected, TSPs significantly disturb the expression and activation of caspases, suggesting that TSPs may regulate apoptosis by acting on the primary drivers of apoptotic cell death. Considering that caspases are regulated by various cytokines in a variety of physiological and pathological conditions, such as inflammation. Whether TSPs have an effect on cell death by middle pathway or directly on apoptosis-related factors needs further investigation. Taken together, our results suggested that TSPs intervention exerts a beneficial role in the gut homeostasis.

The gut barrier prevents excessive interaction of living gut bacteria and microbiome components with the immune system (38). Therefore, accelerating the mucosal healing process and promoting epithelial repair are important goals in the treatment of IBD (39). By increasing the expression of tight junction proteins, protecting the intestinal barrier is considered to be one of the key mechanisms to inhibit the pathogenesis of colitis (40). Here we show that oral administration of TSPs dramatically reverses the decrease of ZO-1 and occluding expression, which was typical of the intestinal epithelium of mice with colitis. According to previous study, genetically modified mice demonstrated that ZO-1 is essential for epithelial repair (41). And promoting occludin expression had proven to be an effective treatment for experimental colitis (42). Therefore, this research illustrated that targeting the tight junction proteins contribute to prevent the inflammatory cascade, which suggested TSPs may improve DSS-induced colonic inflammatory response *via* enhancing intestinal barrier function.

Protein hydrolysate has recently attracted a lot of interest because of its biological activities, which may have the ability to improve human health and reduce illness risk (43, 44). It is well known that the sequence of amino acids governs the different activities of bioactive peptides (7). To explore the peptides composition of TSPs, we used the LC-MS/MS to ascertain the peptides’ sequences. Consistent with reported studies, we found that the majority of these peptides include less than 20 amino acids, and on which more effective bioactivity is predicated (20, 45). Furthermore, we have demonstrated both *in vitro* and *in vivo* that TSPs can reduce inflammatory responses to foreign stimuli. Considering that the peptide bioavailability and associated mechanisms have not yet been entirely elucidated and oral peptides have been reported to be entirely absorbed by intestinal cells under normal conditions and detected in the target tissues where they exhibit biological activities (46), we hypothesized that TSPs may function by reaching the colon through the systemic circulation. Although there is a limitation here that it needs to be proved by more rigorous experiments. This result still strongly supports the speculation that consumption of TSPs maybe has the great potential to impact on other systemic diseases. Thus, TSPs are extremely promising ingredients for functional foods and nutraceuticals. However, in this study, the key peptides of TSPs which play crucial role in attenuating DSS induced colitis need further investigated.

Overall, our findings for the first time indicated that bioactive peptides derived from tilapia skin play a vital role in the alleviation of colitis in mice. *In vitro* and *in vivo* experiments had shown that it may work by directly inhibiting inflammation and apoptosis in the colon epithelium, as mediated by inhibiting the overexpression of inflammatory cytokines and activation of caspase-3 and caspase-8, which could be the main reason for TSPs being so effective as a therapeutic agent. Collectively, our research concluded that TSPs promise as a dietary-based safe and effective alternative therapy for treatment of colon inflammation. More researches are needed in the future to find out the exact peptide sequences and probable receptors for TSPs that are involved in these anti-inflammatory responses.

## Conclusion

To sum up, our study first proposed the therapeutic effects of marine active peptides derived from tilapia skin on DSS-induced colitis. The composition and activity of bioactive peptides in TSPs were characterized and predicted. Mechanistically, TSPs are demonstrated to protect against foreign stimuli by mainly through enhancing the epithelial barrier function, lowering the release of inflammatory cytokines, and suppressing apoptosis. In conclusion, these findings provide new insights into marine peptides for relieving symptoms of colitis and provide new therapeutic strategies for the treatment of IBD and other inflammation-related diseases.

## Data availability statement

The original contributions presented in this study are included in the article/supplementary material, further inquiries can be directed to the corresponding author/s.

## Ethics statement

The animal study was reviewed and approved by Animal Ethics Committee of Shandong University (SYXK: 20190005).

## Author contributions

JG completed all experiments, analyzed the data, and wrote the first draft. DZ and XW supervised the experimental design and revised the manuscript. YX, BL, and CL supervised the experiments. XZ and LL designed the experiments and revised the manuscript. XZ provided the financial support. All authors contributed to the article and approved the submitted version.

## Footnotes

1
http://bioware.ucd.ie/~compass/biowareweb/Server_pages/peptideranker.php
